# Loss of Niemann-Pick C1 or C2 Protein Results in Similar Biochemical Changes Suggesting That These Proteins Function in a Common Lysosomal Pathway

**DOI:** 10.1371/journal.pone.0023677

**Published:** 2011-08-24

**Authors:** Sayali S. Dixit, Michel Jadot, Istvan Sohar, David E. Sleat, Ann M. Stock, Peter Lobel

**Affiliations:** 1 Center for Advanced Biotechnology and Medicine, Piscataway, New Jersey, United States of America; 2 Graduate School of Biomedical Sciences, University of Medicine and Dentistry of New Jersey–Robert Wood Johnson Medical School (UMDNJ–RWJMS), Piscataway, New Jersey, United States of America; 3 Department of Biochemistry, UMDNJ–RWJMS, Piscataway, New Jersey, United States of America; 4 Laboratoire de Chimie Physiologique, Namur Research Institute for Life Sciences and Facultés Universitaires Notre-Dame de la Paix, Namur, Belgium; 5 Department of Pharmacology, UMDNJ–RWJMS, Piscataway, New Jersey, United States of America; Université de Genève, Switzerland

## Abstract

Niemann-Pick Type C (NPC) disease is a lysosomal storage disorder characterized by accumulation of unesterified cholesterol and other lipids in the endolysosomal system. NPC disease results from a defect in either of two distinct cholesterol-binding proteins: a transmembrane protein, NPC1, and a small soluble protein, NPC2. NPC1 and NPC2 are thought to function closely in the export of lysosomal cholesterol with both proteins binding cholesterol in vitro but they may have unrelated lysosomal roles. To investigate this possibility, we compared biochemical consequences of the loss of either protein. Analyses of lysosome-enriched subcellular fractions from brain and liver revealed similar decreases in buoyant densities of lysosomes from NPC1 or NPC2 deficient mice compared to controls. The subcellular distribution of both proteins was similar and paralleled a lysosomal marker. In liver, absence of either NPC1 or NPC2 resulted in similar alterations in the carbohydrate processing of the lysosomal protease, tripeptidyl peptidase I. These results highlight biochemical alterations in the lysosomal system of the NPC-mutant mice that appear secondary to lipid storage. In addition, the similarity in biochemical phenotypes resulting from either NPC1 or NPC2 deficiency supports models in which the function of these two proteins within lysosomes are linked closely.

## Introduction

Niemann Pick Type C (NPC) disease is a neurovisceral lysosomal storage disease that results from mutations in either of two distinct genes: *NPC1* (accounting for ∼95% of cases) or *NPC2* (∼5% of cases). Diseases arising from mutations in either gene are clinically indistinguishable and are characterized by an accumulation of cholesterol and other lipids within the lysosomes of affected individuals (reviewed in [1], [2]). *NPC1* encodes the endolysosomal membrane protein NPC1, a 1278 amino acid glycoprotein with 13 transmembrane segments and a sterol-sensing domain [3] that binds sterols including cholesterol [4], [5], [6]. *NPC2* encodes a 132 amino-acid soluble glycoprotein, NPC2, that primarily localizes to lysosomes [7], [8] and which also binds cholesterol and other sterols [9], [10], [11], [12].

The precise interactions between NPC1 and NPC2 that are required for the lysosomal export of cholesterol have been the focus of considerable attention. Genetic studies have demonstrated that the loss of NPC1, NPC2 or both proteins in mice results in essentially identical phenotypes, suggesting that NPC1 and NPC2 function in concert [13]. In addition, recent biochemical studies indicate that NPC2 can facilitate the intermembrane transfer of cholesterol [14], [15] and can transfer it to NPC1 [16], [17], [18]. The lipid environment can affect NPC2-mediated lipid transfer, with lysosomal phospholipid lyso-bisphosphatidic acid and ceramide accelerating transfer [15], [19] and sphingomyelin inhibiting transfer [19] (reviewed in [20], [21]). Together, these observations provide a basis for a model in which NPC2 transports cholesterol from inner lysosomal membrane structures or microaggregates directly to a sterol binding site in the N-terminal domain of NPC1, which in turn is required for its subsequent egress from the lysosome [1], [22]. This model would appear to require NPC1 and NPC2 to colocalize to some extent. NPC2 clearly resides within the lysosome [7]. The subcellular location of NPC1 is less clear but it is generally thought to reside within the late endosome [23] and the lysosome [24] (reviewed in [25]).

In addition to their intricately-linked roles in cholesterol transport, it is possible that NPC1 and NPC2 may also have other, unrelated functions within the lysosome [26]. In this study, we have investigated this possibility by comparing the biochemical consequences of the loss of each protein in the respective mutant mice. The rationale underlying this approach is that if the NPC1 and NPC2 have different functions within the lysosome then in the absence of either protein, there may be differences in the biochemical phenotype.

## Results and Discussion

### Survival of BALB/c NPC mutants

**Figure 1:**
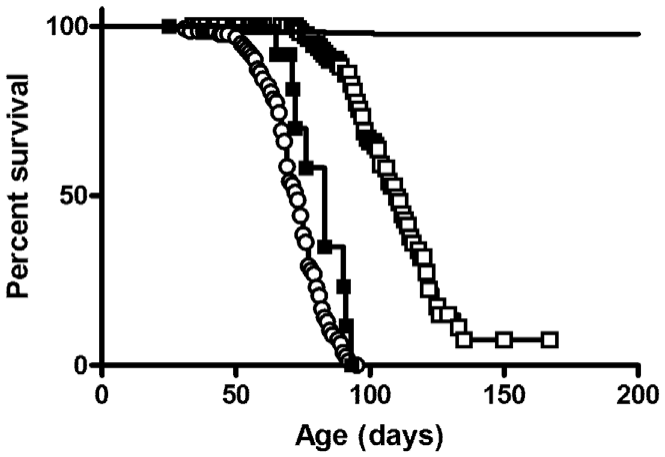
Survival of the NPC-mutant mice. Wild-type (solid line, n = 381 animals, 8 deaths), *Npc1* mutants (○, n = 134 animals, 91 deaths), *Npc2* mutants (□, n = 150 animals, 66 deaths) and *Npc1/Npc2* double mutants (▪, n = 15 animals, 9 deaths) were in a BALB/c genetic background.

While the spontaneous *Npc1* mutant mouse appears to be a null [27], gene targeting of *Npc2* resulted in a mouse that expresses up to 4% of normal levels of functional NPC2 protein [13], thus the *Npc2* mouse model is technically a hypomorph. Previous comparison of survival of mutant mice indicated that the phenotype of the *Npc2* mutant was extremely similar but attenuated compared to the *Npc1* or *Npc1*/*Npc2* double mutants [13]. This attenuation likely reflected residual NPC2 expression thus epistasis arguments suggested that both proteins functioned in a common pathway. However, interpretation of these results was complicated by the fact that these studies were conducted using mutants with an indeterminate (mixed C57BL/6, 129SvEv and BALB/c) genetic background and that strain background could have a significant effect on survival. To exclude possible strain effects, we introduced the *Npc2* mutant allele into a BALB/c background by backcrossing and compared the phenotype of these congenic animals with the *Npc1* mutant in the same strain (Fig.1). We find that mice lacking NPC1 have the shortest life span with median survival 73 days (95% confidence interval (c.i.), 69 to 75 days). Survival of NPC2-deficient mice was significantly longer than that of *Npc1* single mutants (log rank test p<0.0001), with median survival 110 days (95% c.i. 106 to 116 days). Survival of mutant mice with deficiencies in both NPC1 and NPC2 was similar to those lacking NPC1 alone, with median survival 83 days (95% c.i. 72 to >83 days). These data verify that the phenotype of the *Npc2* mutant is attenuated compared to the *Npc1* mutant and eliminates the possibility that the difference in survival could be related to strain effects.

### Effect of the loss of NPC1 and NPC2 on biophysical properties of liver and brain organelles

NPC disease is associated with a block in the intracellular trafficking of unesterified cholesterol and other lipids, leading to their accumulation within the endosomal/lysosomal system. If both NPC1 and NPC2 proteins function in a common pathway, deficiencies in either should produce similar effects on the biophysical properties of lysosomes. Previously, we conducted subcellular fractionation of brains from symptomatic *Npc2* mutants and found significant decrease in the buoyant density of the lysosome but not in other cellular organelles [28]. Others have found a decrease in the buoyant density of lysosomes in the liver, spleen and lung from symptomatic *Npc1*-mutant animals [29], [30]. Given the complex pathological cascades that are associated with disease progression, we used presymptomatic animals to investigate the effect of NPC1 and NPC2 deficiencies on the properties of brain and liver organelles.

**Figure 2:**
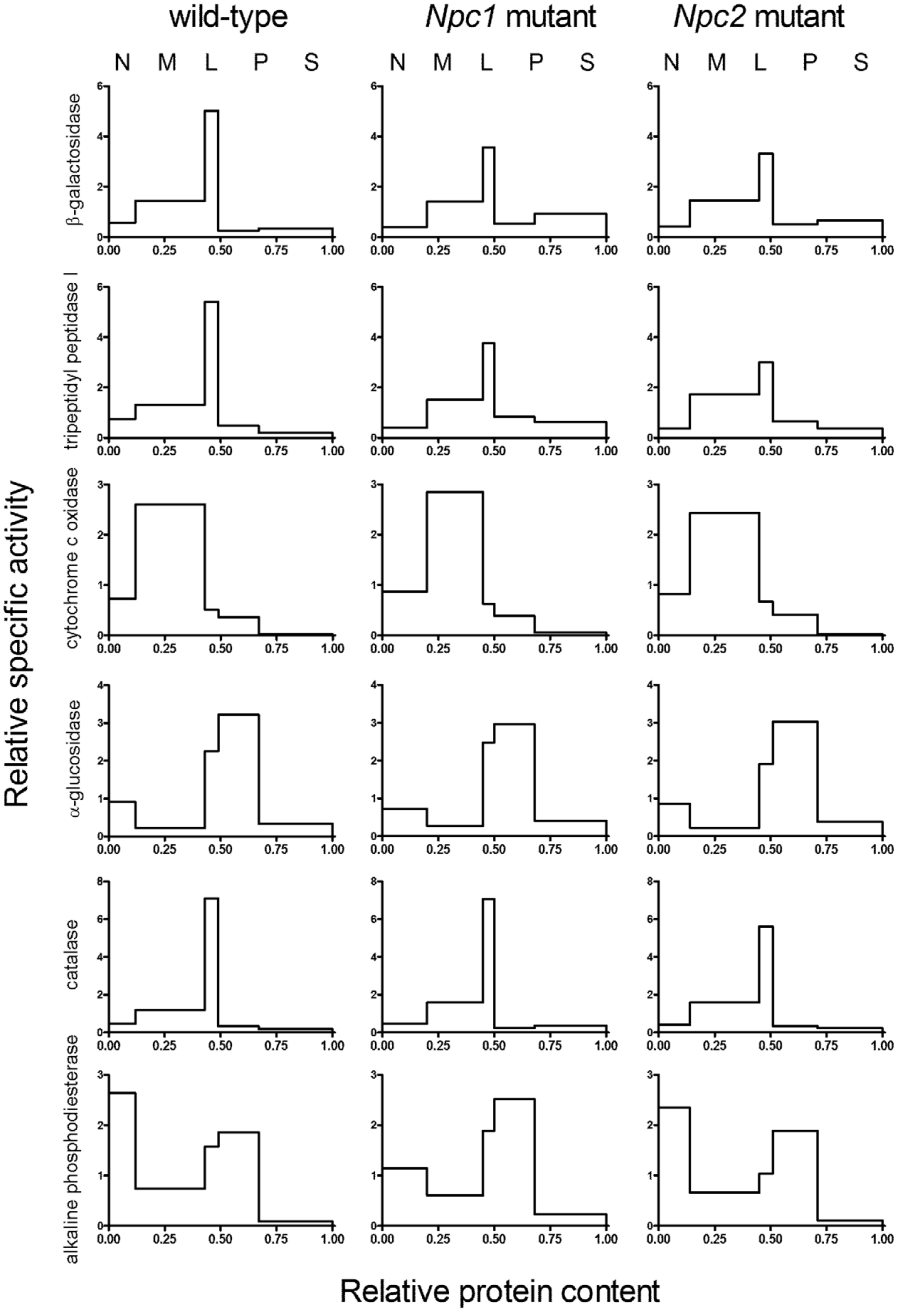
Relative distributions of marker enzymes in differential centrifugation fractions of livers of wild-type and NPC-mutant mice. Subcellular fractions were generated from livers of wild-type and *Npc1*- and *Npc2*-mutant mice by differential centrifugation. Fractions were analyzed for the following marker enzymes: β-galactosidase (lysosome), tripeptidyl peptidase I (lysosome), cytochrome c oxidase (mitochondria), α-glucosidase II (endoplasmic reticulum), catalase (peroxisome) and alkaline phosphodiesterase (plasma membrane). Ordinate, relative specific activity of each fraction (proportion of total recovered activity/proportion of total recovered protein). Abscissa, relative protein content of fraction (cumulative from left to right). Areas are proportional to the total activity in any given fraction. Fractions are nuclear (N), heavy mitochondrial (M), light mitochondrial (L), microsomal (P) and high-speed supernatant (S).

**Figure 3:**
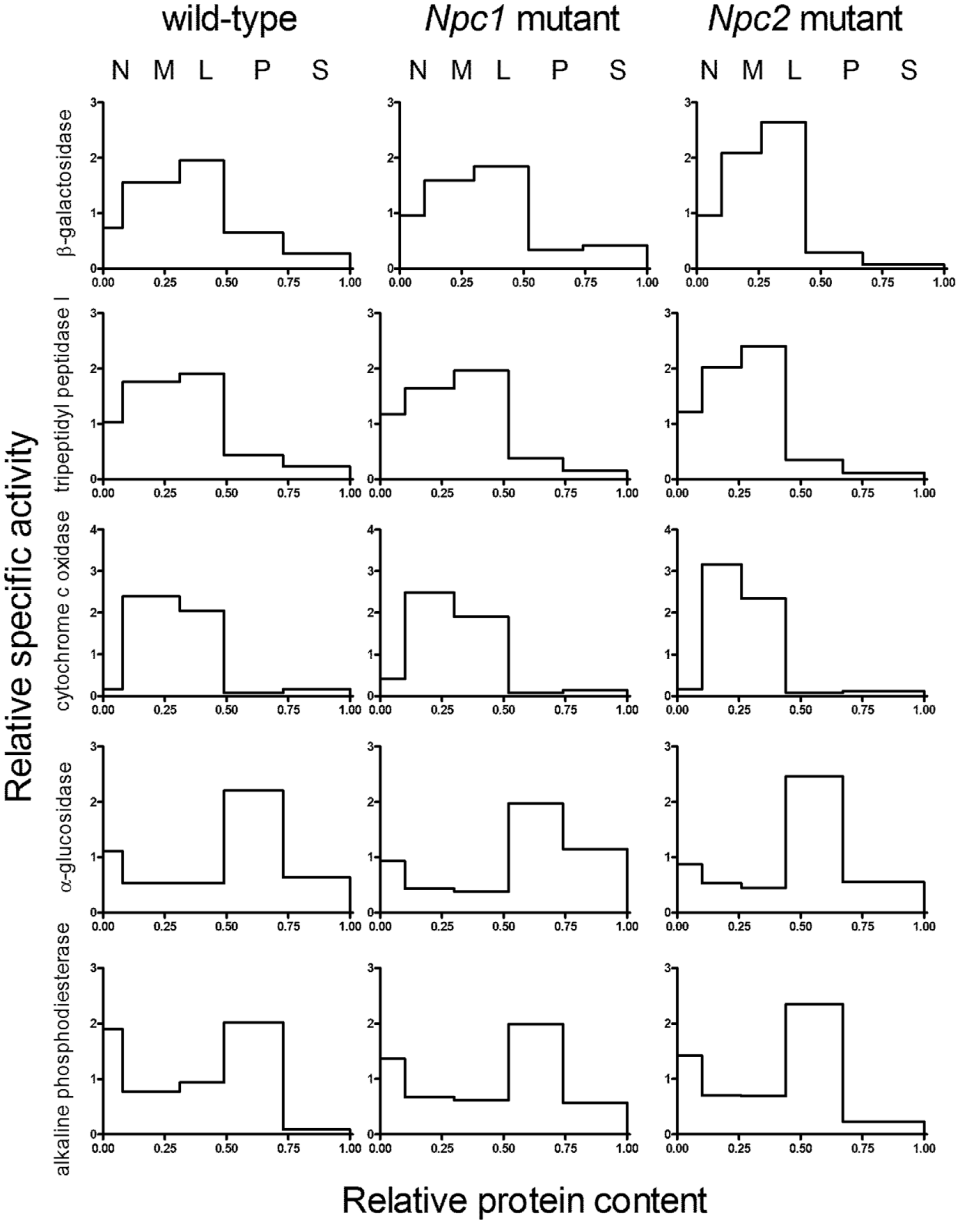
Relative distributions of marker enzymes in differential centrifugation fractions of brains of wild-type and NPC-mutant mice. Details are as in Figure 2. Note that catalase activity was very low in brain and is not shown.

Lysosomal enzyme activities β-galactosidase and tripeptidyl peptidase I (TPP1) , as well as markers from other organelles including mitochondria (cytochrome c oxidase), endoplasmic reticulum (α-glucosidase II), peroxisomes (catalase) and plasma membrane (alkaline phosphodiesterase) were measured in differential centrifugation fractions isolated using the classical five fraction scheme [31]. Fractions are nuclear (N), heavy mitochondrial (M), light mitochondrial (L), microsomal (P) and high-speed supernatant (S). In wild-type liver, as expected, the highest specific activity for each lysosomal enzyme was found in the L fraction with the majority of the activity being in the M fraction (Fig. 2). (Note in this presentation, for each fraction, the height of the bar represents the enrichment of the specific activity (activity/protein) of interest compared to the initial homogenate while the area of the bar represents the proportion of total activity found in that fraction). Relative distributions of the lysosomal markers in the differential centrifugation fractions from livers of the NPC-mutant mice were similar to those of wild-type mice but there were some differences. There was a consistent decrease in the enrichment of the lysosomal markers in the L fraction of NPC-mutant livers with a concomitant increase in the S fraction (based on 4 independent experiments). The pathogenic implications of these observations are difficult to evaluate as the appearance of luminal lysosomal hydrolases in the S fraction most likely occurs due to rupture of large, lipid-laden lysosomes that occurs when homogenizing NPC-deficient liver [30]. Relative distributions of the markers for other organelles were similar in the differential centrifugation fractions from NPC-mutant livers (Fig. 2). In the brain, the absence of either NPC1 or NPC2 had no significant effect on the relative distribution of the lysosomal markers (Fig. 3), as noted previously for NPC2 [28]. This differs from other reports [32] but may reflect the fact that we analyze the complete brain, rather than discrete regions. Alternatively, this may be due to differences in preparing the organelle-enriched subcellular fractions.

**Figure 4:**
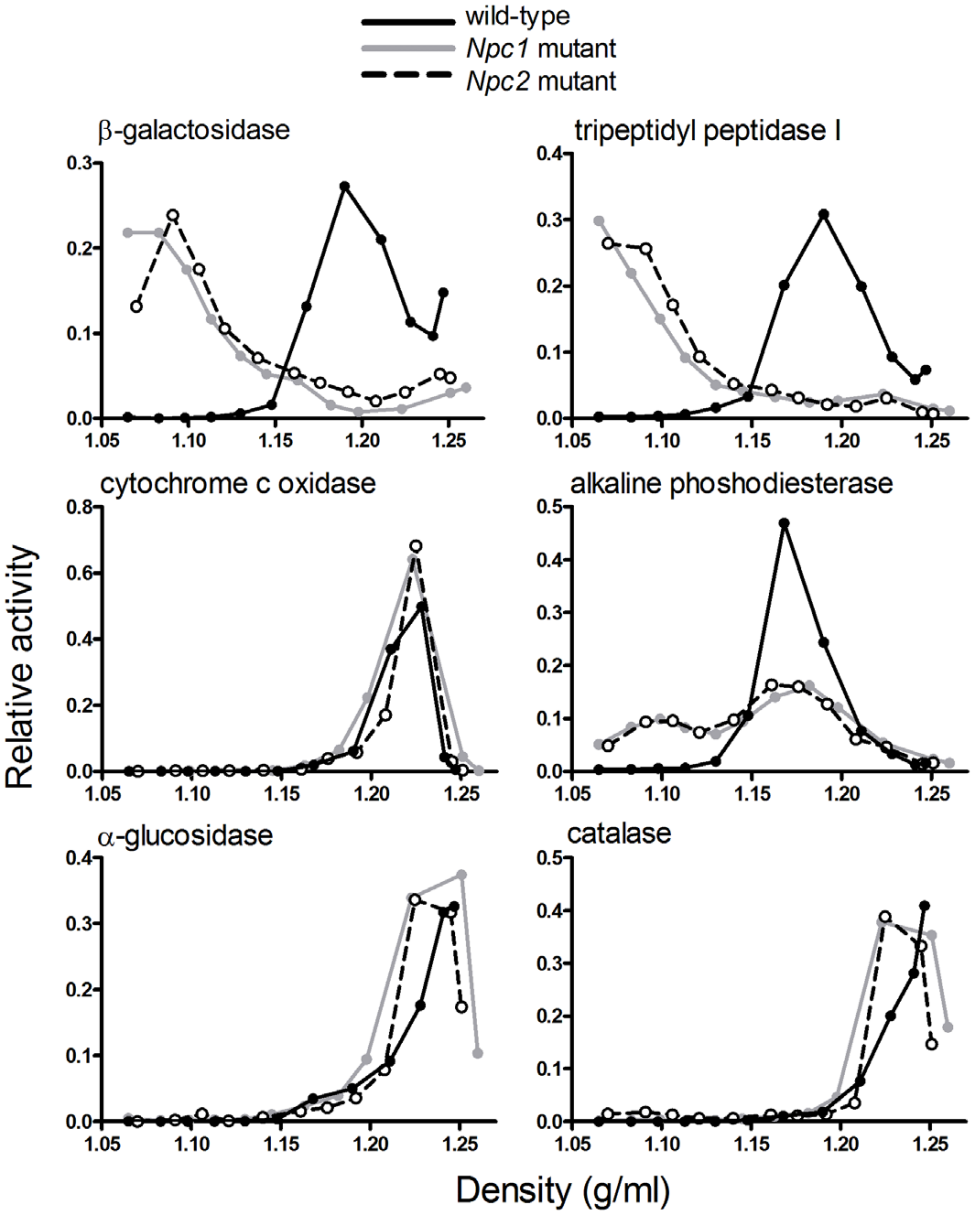
Enzyme distribution in sucrose density gradient fractions from livers of wild-type and NPC-mutant mice. ML differential centrifugation fractions were prepared from livers of wild-type (black solid line), *Npc1* mutant (grey solid line) and *Npc2* mutant (black dotted line) mice and were then fractionated on sucrose density gradients. Relative enzyme activities (proportion in each fraction of total recovered activity) are plotted against densities of the respective fractions.

**Figure 5:**
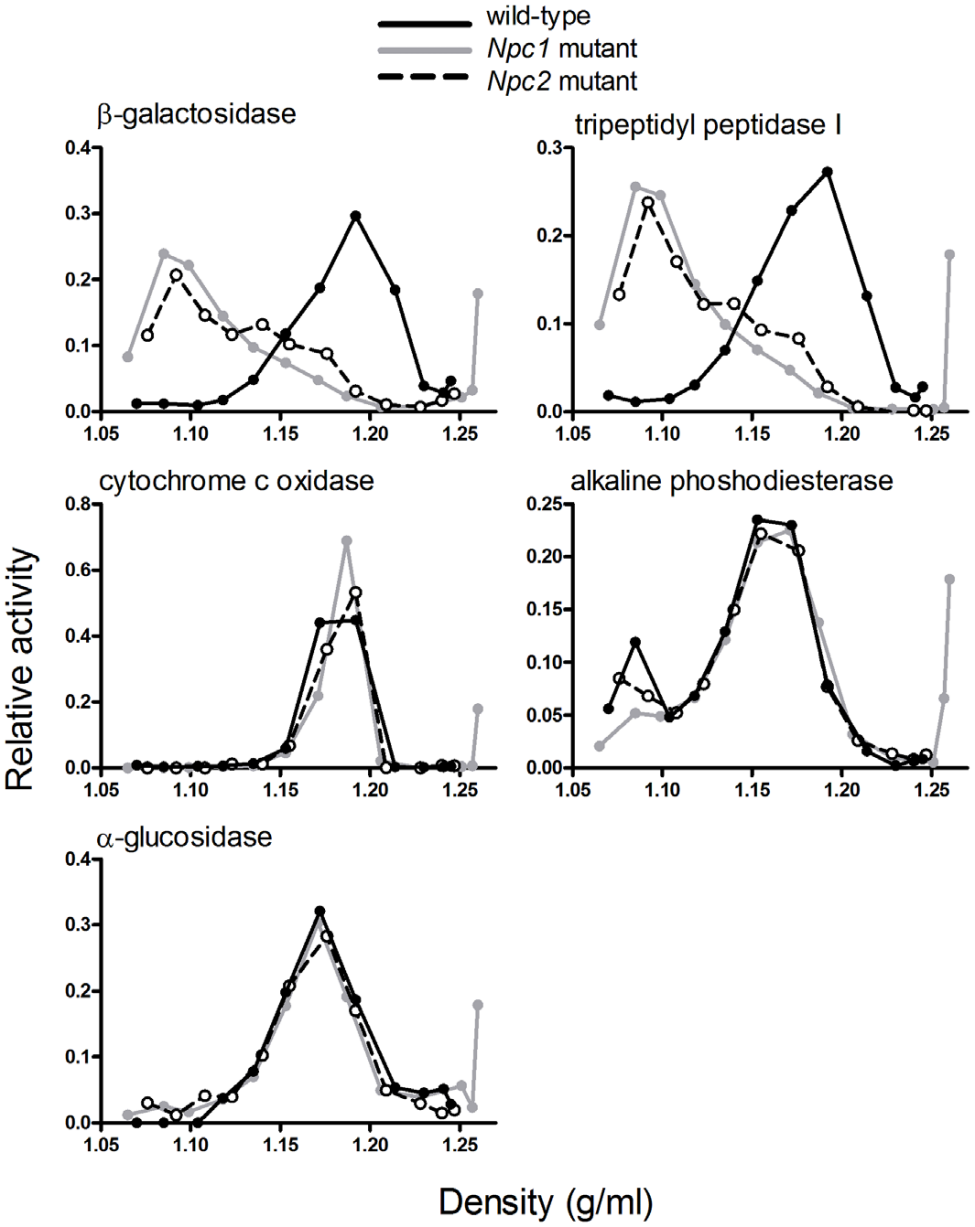
Enzyme distribution in sucrose density gradient fractions from brains of wild-type and NPC-mutant mice. Details as in Figure 4.

We performed sucrose density gradient centrifugation on a combined ML differential centrifugation fraction prepared from the liver (Fig. 4) and the brain (Fig. 5) and determined distributions of lysosomes and other cellular organelles by measuring the marker enzymes. In both the liver and the brain, distributions of the lysosomal markers revealed a dramatic shift in the buoyant density of lysosomes prepared from the mutants compared to those of control wild-type mice (Figs. 4 and 5). In the liver, both *Npc1* and *Npc2* mutants exhibited a change in the appearance of alkaline phosphodiesterase, compared to wild-type animals, with some of the activity shifting to a lower density. While this enzyme is typically regarded as a plasma membrane marker, others have reported some localization within the lysosome [33]. In contrast, analysis of other marker activities indicated that the density of liver and brain mitochondria and endoplasmic reticulum, brain plasma membrane, and liver peroxisomes did not appear to be affected by the loss of either NPC protein (Figs. 4 and 5). Most importantly, while both *Npc1* and *Npc2* mutants exhibited marked differences compared to controls, there were no appreciable differences in the organelle properties of both mutants.

### Subcellular distribution of NPC1 and NPC2 in the NPC-mutant mice

**Figure 6:**
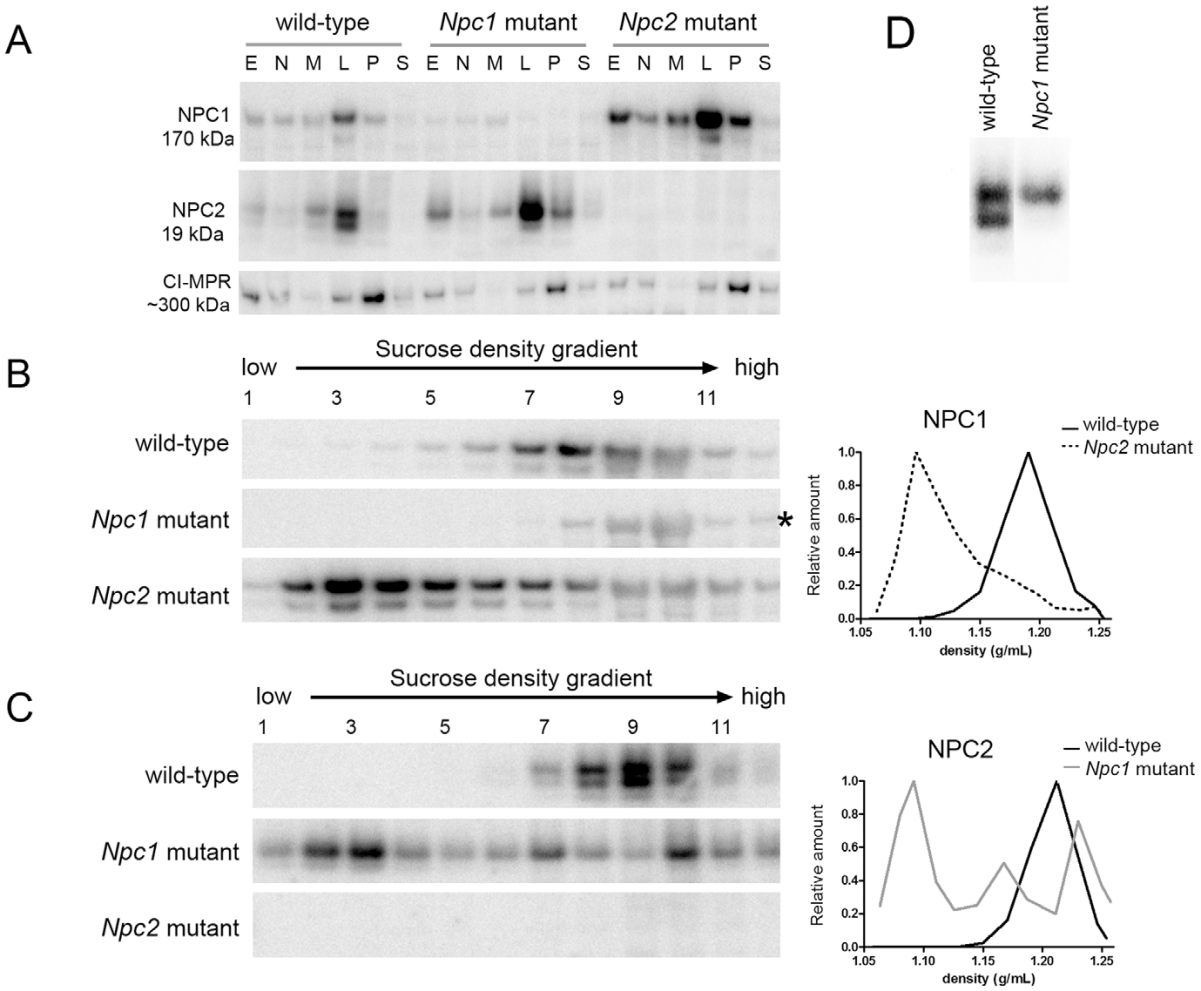
Distribution of NPC1 and NPC2 in subcellular fractions of livers of wild-type and NPC-mutant mice. **A**. Equivalent amounts of protein of the indicated differential centrifugation fractions were fractionated by SDS-PAGE gels and NPC1, NPC2 and CI-MPR were detected by western blotting. ML differential centrifugation fractions of livers from wild-type, *Npc1*- and *Npc2*-mutant mice were further fractionated on sucrose density gradients. Volume equivalents of the gradient fractions were run on SDS-PAGE gels and were probed for NPC1 (**B**) and NPC2 (**C**) by immunoblotting. Note that a non-specific band is detected that migrates in approximately the same position as NPC1 (indicated by asterisk in Panel B). This does obscure NPC1 detection, particularly in Fractions 9 and 10 but the shift in density upon loss of NPC2 is clear. However, for this reason, in quantifying distribution across the gradients of both NPC1 and NPC2, the corresponding lanes from each respective knockout were subtracted as background. Data are normalized to maximum value. **D**. Grey-scale matched and magnified inset of NPC2 detected in the L differential fraction from wild-type and *Npc1*-mutant mouse liver.

**Figure 7:**
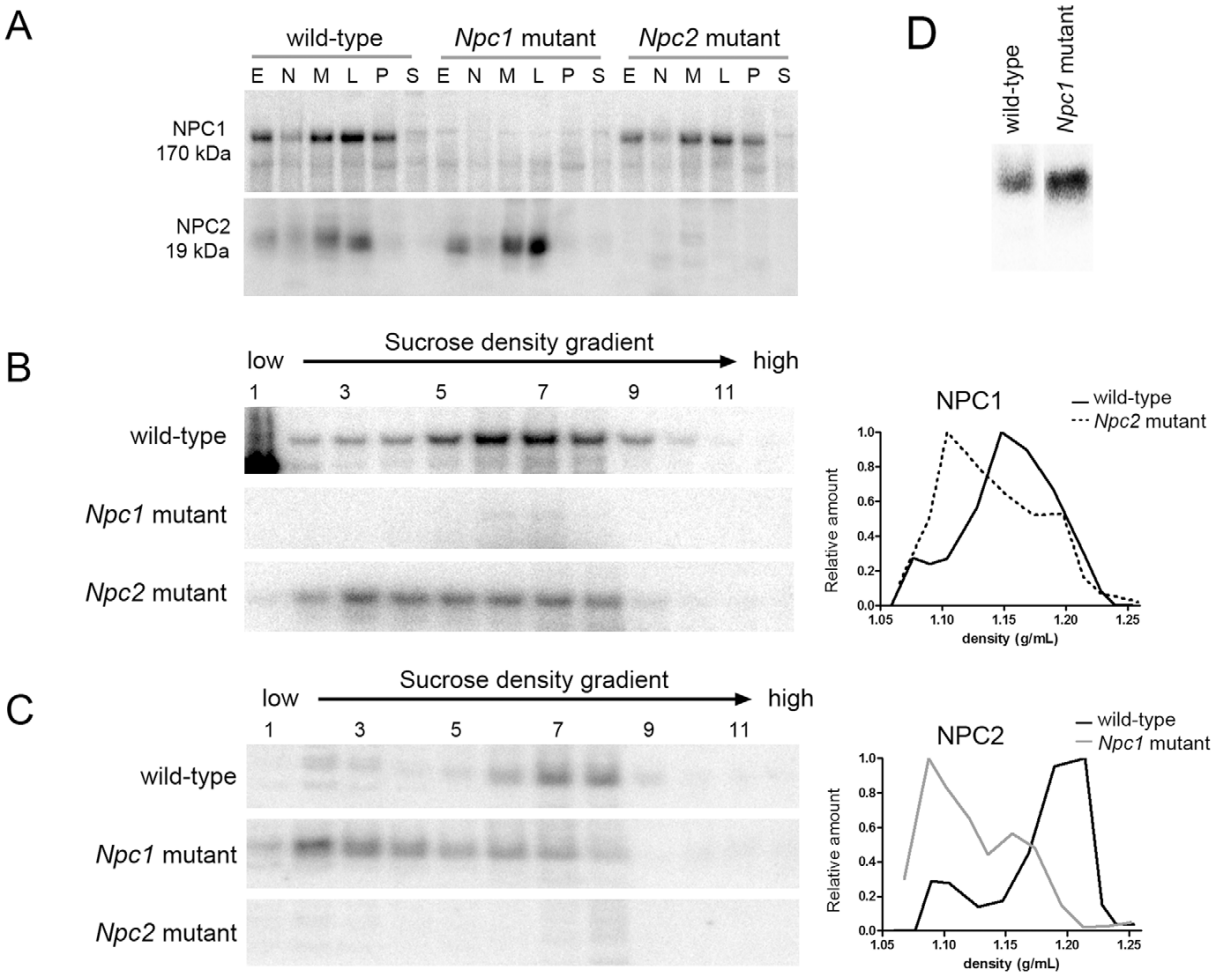
Distribution of NPC1 and NPC2 in subcellular fractions of brains of wild-type and the NPC-mutant mice. Details are as in Figure 6 except Panel D is not grey-scale matched.

We investigated the subcellular distribution of NPC1 and NPC2 proteins in the NPC-mutant mice by immunodetection in both the differential centrifugation fractions and sucrose density gradients of ML fractions prepared from liver (Fig. 6) and brain (Fig. 7). In the differential centrifugation fractions, distribution of the membrane-associated NPC1 was unaffected by the absence of NPC2, and vice versa, in either the liver or the brain (Fig. 6, Panel A and Fig. 7, Panel A). Immunoblotting indicated that the distribution of the cation-independent mannose 6-phosphate receptor (CI-MPR), which traffics between the trans-Golgi network, plasma membrane, and early and late endosomes [34] appeared unaltered by the absence of NPC1 or NPC2.

**Figure 8:**
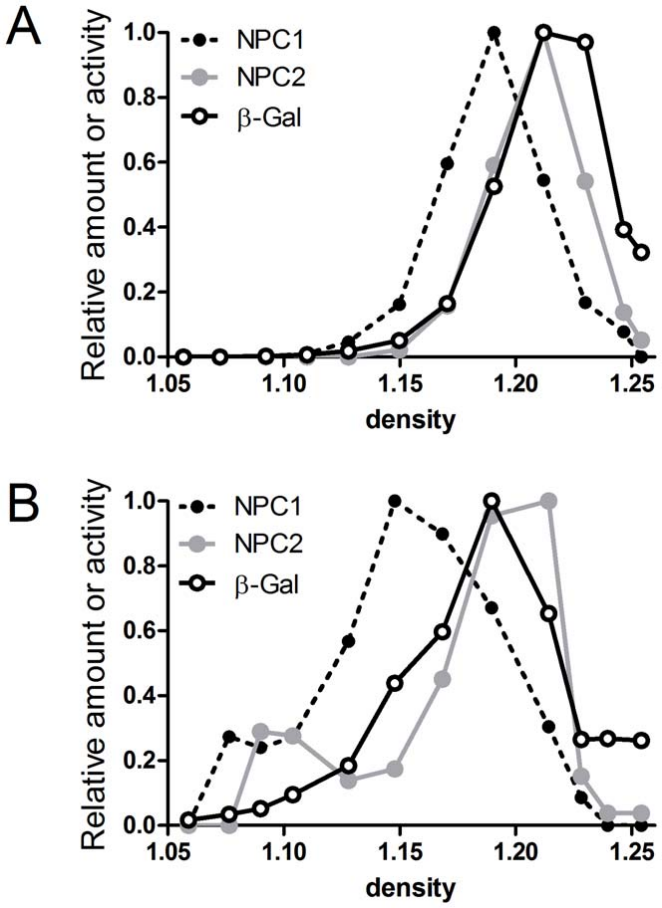
Distribution of NPC1 and NPC2 in sucrose density gradients of ML differential fractions from wild-type mice. NPC1 and NPC2 were quantitated by densitometry from data shown in Figs. 6 and 7. Also shown is the distribution of the lysosomal marker, β-galactosidase. **A**, liver; **B**, brain. Data are normalized to fraction of maximum value.

Distribution of NPC1 and NPC2 in the NPC-mutant mice was also investigated by sucrose density gradient centrifugation (Fig. 6, Panels B and C; Fig. 7, Panels B and C). In wild-type mice, both NPC1 and NPC2 colocalized to high-density gradient fractions (liver fractions 7–12 (Fig. 6), brain fractions 5–8 (Fig. 7)). However, in the mutant mouse livers and brains, NPC1 and NPC2 were distributed across the gradient with peaks at a low-density region (liver fractions 1–5 (Fig. 6), brain fractions 2–5 (Fig.7)). These data are consistent with NPC1 and NPC2 residing in a lysosomal compartment that is similarly affected by the lipidosis induced by a NPC1 or NPC2 deficiency. However, it is interesting to note that in both wild-type brain and liver, the distribution of NPC1 appears to be slightly shifted towards lower density fractions compared to NPC2 and β-galactosidase, which essentially cofractionate (Fig. 8). This may indicate a subtle difference in the subcellular localization of NPC1 and NPC2. Alternatively, this may reflect intrinsic differences in the density distribution of lysosomal membrane proteins (e.g., NPC1) versus soluble proteins (e.g., NPC2) [35]. For example, in a lysosomal population of heterogeneous size, the specific activity (i.e., activity per unit protein) of membrane proteins will decrease with increasing lysosome size, reflecting the decreasing ratio between membrane surface area and volume of the lumen (which contains the bulk of protein). In the absence of lipidoses, as the membrane surface area to volume ratio decreases, the density of the lysosome will increase as the relative proportion of membrane lipids is decreased. In effect, this will increase the relative specific activity of membrane proteins in the smaller, lower density particles.

### Glycosylation of lysosomal proteins in the NPC-mutant mice

Most newly synthesized soluble lysosomal proteins are glycoproteins that are transported to lysosomes by virtue of a specific targeting signal, mannose-6-phosphate. The N-linked glycans undergo considerable processing, both during transit through the ER and Golgi as well as after delivery to lysosomes. In the lysosome, sugars on these proteins, including NPC2, are processed by other lysosomal enzymes e.g., α-mannosidase [36], [37], [38] but are not completely removed. Mature lysosomal proteins exhibit signature glycosylation patterns that can differ in different cell types and tissues. When analyzed by SDS-PAGE, murine liver NPC2 normally migrates as several bands that likely result from heterogeneity in its single N-linked oligosaccharide [39]. It has been reported that the glycosylation of NPC2, as reflected by electrophoretic mobility, is altered in NPC1-deficient mouse liver compared to wild-type controls, with a shift towards a higher molecular weight glycosylated form. This alteration in carbohydrate processing was suggested to be specific to NPC2 rather than reflecting a widespread cellular defect. This led to the proposal that deficiencies in NPC1 resulted in a malfunction of NPC2 as reflected by aberrant glycosylation [39], and that this was the basis for the cholesterol storage observed in NPC1 disease.

**Figure 9:**
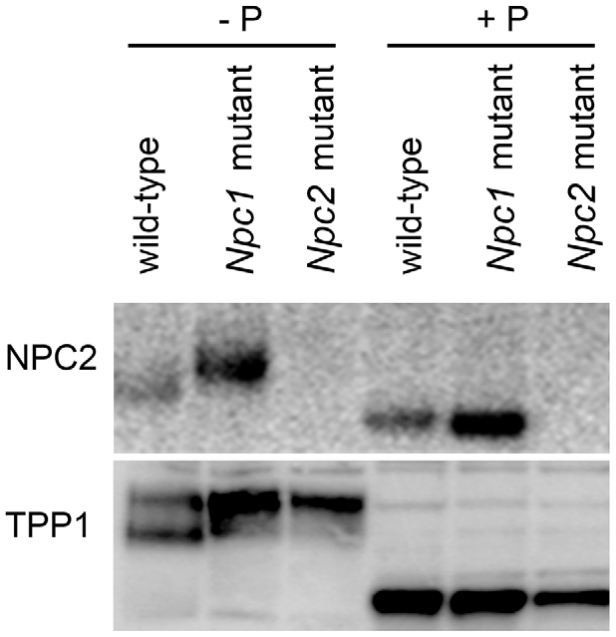
Glycosylation of lysosomal proteins in the liver of wild-type and NPC-mutant mice. Protein equivalents of the L differential centrifugation fractions were fractionated on SDS-PAGE gels and NPC2 and TPP1 were visualized by immunoblotting. Where indicated (+P), samples were digested with PNGase F prior to SDS-PAGE.

We also find that the appearance of NPC2 is different in wild-type and NPC1-deficient liver. Fig. 6, Panel D shows magnified immunoblots of NPC2 detected in the L differential centrifugation fractions from wild-type and *Npc1*-mutant mice. (Given that NPC2 is expressed more abundantly in the *Npc1*-mutant mice, the two panels have been grey-scale matched to allow qualitative comparison of the NPC2 isoforms). In the wild-type mouse, NPC2 is detected as a doublet of apparent *M*_r_ of 18 and 20 kDa but in the *Npc1*-mutant, it is detected only as the higher weight isoform. Treatment of liver extracts from both wild-type and *Npc1*-mutant mice with PNGase F to remove N-linked oligosaccharides resulted in a collapse of all NPC2 isoforms to an identical, lower molecular weight species, indicating that the changes in the mass of NPC2 in the absence of NPC1 arise from differences in N-linked glycosylation (Fig. 9). These findings are consistent with previous observations from liver [39]. However, in brain, we find no apparent changes in the glycosylation of NPC2 in the absence of NPC1 (Fig. 7). Given that neurodegeneration is a prominent feature of NPC disease, these results do not appear to be consistent with aberrant glycosylation of NPC2 being associated with disease.

To investigate the specificity of these changes in glycosylation, we also examined TPP1, another soluble lysosomal protein, in liver from both NPC1- and NPC2-deficient mice (Fig. 9). In wild-type liver, TPP1 migrates on SDS-PAGE as a heterogeneous series of bands of ∼45–48 kDa. However, we find that in the absence of either NPC1 or NPC2, TPP1 now migrates predominantly as a single band that corresponds to the higher mass isoforms detected in the wild-type liver. PNGase F treatment shifts all the bands to a lower molecular weight species (Fig. 9). These results indicate that alterations in glycosylation observed in liver in the absence of NPC1 are not specific to the NPC2 protein. Furthermore, alterations in the glycosylation of TPP1 are observed in the absence of either NPC1 or NPC2. Taken together, these results indicate that the alterations in the glycosylation of soluble lysosomal proteins in liver are a secondary effect of NPC disease resulting from either *Npc1* or *Npc2* mutations. Thus, altered processing of NPC2 in the absence of NPC1 is unlikely to be of pathogenic consequence. This conclusion is supported by the earlier finding that all glycoforms of NPC2 bound cholesterol and could ameliorate the cholesterol storage phenotype of fibroblasts from NPC2 patients [10], indicating that heterogeneity in glycosylation appears not to be directly related to its function.

### α-Mannosidase activity in the NPC-mutant mice

**Figure 10:**
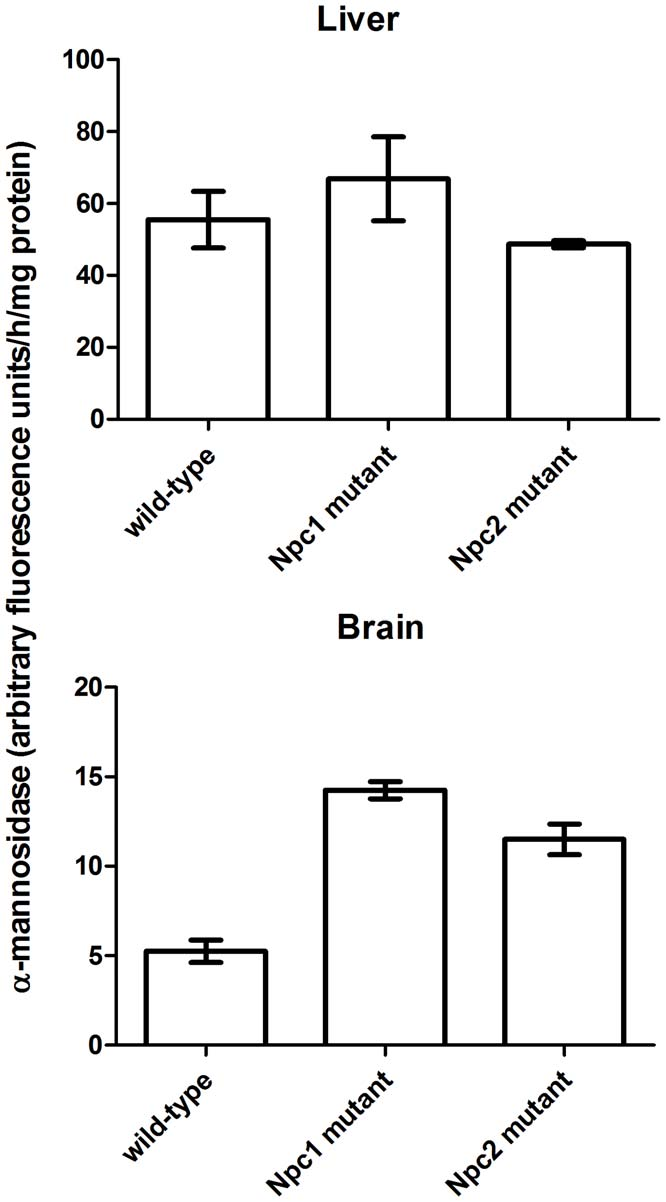
α-Mannosidase activity in livers and brains of wild-type and NPC-mutant mice. α-Mannosidase activity was normalized to protein and was measured from 4 mice per group. Error bars represent standard error of the mean. Using Dunnett's multiple comparison test, α-mannosidase activity in brain was significantly (p<0.001) greater in mice lacking either NPC1 or NPC2 compared to wild-type. No significant differences were detected in liver.

Recent studies indicate that the loss of α-mannosidase activity results in defective processing of N-linked oligosaccharides with increased levels of glycosylation on NPC2 and other lysosomal proteins [37], [38]. We therefore considered the possibility that the altered glycosylation of NPC2 and TPP1 in liver in the absence of NPC1 might reflect a decrease in the activity of α-mannosidase that is secondary to the primary defect in cholesterol transport. We find that α-mannosidase activity is increased in brain in the absence of either NPC1 or NPC2 (Fig. 10), and this is consistent with the normal carbohydrate processing of lysosomal proteins in this organ (Fig. 7). In liver, where deglycosylation of lysosomal proteins is impaired in the absence of NPC1 or NPC2 (Fig. 9), α-mannosidase activity is unaffected by the loss of either protein (Fig. 10). This indicates that a down-regulation of α-mannosidase synthesis is not the cause of impaired deglycosylation. However, the possibility that the accumulation of storage lipids within the lysosome might reduce α-mannosidase activity or limit accessibility to certain substrates, cannot be excluded.

### Concluding Remarks

The aim of this study was to use biochemical tests to determine whether NPC1 and NPC2 might have unrelated functions within the lysosome in addition to their roles in cholesterol transport. Our rationale was that if these proteins function exclusively in cholesterol transport, then the loss of either would be predicted to yield similar phenotypes. Conversely, should NPC1 or NPC2 have a unique function within the lysosome, then we hoped to observe specific differences in the biochemical phenotype in the absence of either protein. We find that NPC1 and NPC2 appear to have considerable overlap in distributions and that a deficiency in either protein elicits similar shifts in the buoyant density of the organelle. We also find that the loss of either NPC1 or NPC2 both result in aberrant carbohydrate processing of another lysosomal protein, TPP1. This indicates that the altered glycosylation of NPC2 observed in the absence of NPC1 [39] is not specific to NPC2 and thus altered glycosylation of NPC2 appears not to be the primary cause of disease as has been suggested elsewhere [39]. These data, along with our previous measurements of storage material and neuropathological analysis of *Npc1* and *Npc2* mutants [13] are entirely consistent with roles for NPC1 and NPC2 in the lysosome in sequential steps of a cellular cholesterol transport pathway.

The model that NPC1 and NPC2 play closely related roles in lysosomal cholesterol transport does not preclude additional specialized extralysosomal roles for these two proteins. For instance, NPC2 is secreted into epididymal fluid [40] and breast milk [41] and could conceivably play a role in sterol transport in such extracellular aqueous environments. NPC2 is also present in bile [42] and its function in this location remains unknown although its presence here appears not to be required for the intestinal uptake of cholesterol [43]. If, like the RND permeases, NPC1 plays a role in trans-bilayer transport of a variety of hydrophobic and amphipathic molecules, it could be potentially be involved in the lysosomal egress of a wider range of substrates than is bound by NPC2. Interestingly, while NPC1 binds cholesterol and oxysterols [4], NPC2 exhibits little or no affinity for the latter [5], [10]. Nonetheless, given that cholesterol is one of the major lipids in all mammalian cells and it maintains a considerable flux through the lysosome, it seems likely that the primary function of both proteins lies in facilitating lysosomal cholesterol egress and that the primary defect in NPC disease is a dysfunction within this pathway.

## Materials and Methods

### Ethics Statement

All experiments and procedures involving live animals were conducted in compliance with protocols approved by the Robert Wood Johnson Medical School Institutional Animal Care and Use Committee (“Animal Models to Investigate Niemann-Pick Type C Disease”, protocols I10-010 and I07-006). All efforts were made to minimize suffering.

### Primary antibodies

A rabbit polyclonal antibody raised against NPC1 was kindly provided by Dr. Daniel S. Ory. Rabbit polyclonal antibodies against NPC2 (affinity purified preparation HL5895), TPP1 (bleed R72/5) [44] and the CI-MPR (affinity purified preparation PL603C1) were generated in-house. Goat anti-rabbit IgG (catalog number R4880) was obtained from Sigma (St. Louis, MO).

### Western blotting

SDS-polyacrylamide gel electrophoresis was conducted using precast Novex bis-Tris gels and running buffers (Invitrogen, Carlsbad, CA) as indicated: NPC2 (non-reducing, 4–12% polyacrylamide, MES buffer), TPP1 (reducing,10% polyacrylamide, MOPS buffer), and NPC1 (reducing, 10% polyacrylamide, MES buffer). Proteins were transferred from the gels to nitrocellulose membranes using a semi-dry transfer unit (Bio-Rad Laboratories, Hercules, CA). Membranes were dried and blocked overnight at 4°C or 1 h at room temperature (RT) in PBS / 0.2% Tween 20 containing either 5% dry fat-free milk (TPP1), 1% BSA (NPC1) or 5% BSA (NPC2). Blocked membranes were incubated overnight at 4°C or 1 h at RT with primary antibody solutions in PBS / 0.2% Tween 20 containing 5% dry fat-free milk (TPP1) or 5% BSA (NPC1, NPC2). Membranes were then washed 3 times with PBS / 0.2% Tween 20 for 10 min at RT then incubated with iodinated goat anti-rabbit IgG for 2 h at RT for radiographic detection. This antibody was iodinated using Iodogen (Pierce) and ^125^I (PerkinElmer, Inc, Waltham, MA). Following incubation, membranes were washed as before, air dried, exposed to phosphor storage screens, and scanned using a Typhoon 9400 scanner (GE Healthcare, Piscataway, NJ). Protein levels were quantitated using ImageQuant 5.2 (GE Healthcare).

### Animals

*Npc1* and *Npc2* mutants have been described previously [13], [27]. The *Npc2* mutant allele was introduced into a BALB/c genetic background by >10 generations of backcrossing to BALB/c mice while the *Npc1* mutation arose from a spontaneous transposon insertion in BALB/c animals [45]. Experimental animals were homozygous with respect to wild-type or mutant alleles. For survival studies, median survival times and 95% confidence intervals were computed using the survival package in R version 2.13.0 (http://www.R-project.org).

### Subcellular fractionation

Four to six week old mice were fasted overnight and then deeply anesthetized by intraperitoneal injection of sodium pentabarbitol and sodium phenytoin (Euthasol, Delmarva Laboratories, Inc., Midlothian, VA) and then perfused with saline. Livers and brains of the animals were removed and resuspended in 2–3 volumes of ice-cold 0.25 M sucrose in a Potter homogenizer. Tissues were homogenized by three passages of a motorized rotating pestle (1500 rpm) from top to bottom of the homogenizer. The resulting homogenates were subjected to a series of centrifugation steps at 4°C to generate the five fractions (see Results) described by de Duve *et al.* [31]. Isopycnic centrifugation on sucrose density gradients was conducted as described [46] with minor modifications. The ML fractions were mixed with 1.32 g/cm^3^ sucrose solution to give final density higher than 1.26 g/cm^3^. These samples were applied at the bottom of sucrose density gradients prepared in 1.05–1.26 g/cm^3^ density range. Tubes were centrifuged at 40,000 RPM for 150 min in a Sorvall SW50.1 rotor and then sliced from the top to the bottom into 12 fractions. Densities of all the fractions were measured by refractometry and samples were stored at −80°C prior to use. Enzyme and protein assays were performed as described previously [28], [44], [47].
